# Predictors of influenza vaccine uptake during the 2009/10 influenza A H1N1v (‘swine flu’) pandemic: Results from five national surveys in the United Kingdom

**DOI:** 10.1016/j.ypmed.2015.12.018

**Published:** 2016-03

**Authors:** You Kyung Julia Han, Susan Michie, Henry W.W. Potts, G. James Rubin

**Affiliations:** a King's College London, Department of Psychological Medicine, Weston Education Centre, Cutcombe Road, London SE5 9RJ, UK; b University College London, Division of Psychology and Language Sciences, London, UK; c University College London, Centre for Health Informatics and Multiprofessional Education, UCL Institute for Health Informatics, London, UK

**Keywords:** Influenza, Vaccination, Pandemic, Adherence, GP, General practitioner [primary care practitioner]

## Abstract

**Objectives:**

To investigate reasons underlying the low uptake of the influenza A H1N1v vaccination in the UK during the 2009/10 pandemic.

**Methods:**

We analysed data from five national telephone surveys conducted in the UK during the latter stages of the pandemic to identify predictors of uptake amongst members of the public offered the vaccine by their primary care physician (*n* = 1320). In addition to demographic variables, participants reported: reasons for declining the vaccination, levels of worry about the risk of catching swine flu, whether too much fuss was being made about the pandemic, whether they or a close friend or relative had had swine flu, how effective they felt the vaccine was, whether they had previously had a seasonal flu vaccination, how well prepared they felt the government was for a pandemic and how satisfied they were with information available about the pandemic. Most participants (*n* = 734, 55.6%) reported being vaccinated against swine flu, compared to 396 who had not been vaccinated and were unlikely to be vaccinated in the future.

**Results:**

The main reasons given for declining vaccination were concerns over the vaccine's safety, and being generally healthy. Controlling for demographic variables, risk factors for not being vaccinated were: being female, not having a long-standing infirmity or illness, not having been vaccinated against seasonal flu in previous years, feeling that too much fuss had been made about the pandemic and believing that the vaccine was ineffective.

**Conclusions:**

Interventions that target these factors may be effective in improving uptake in a future pandemic.

## Highlights

• We analysed data from UK telephone surveys conducted during the 2009/10 pandemic.
• We explored reasons for accepting or declining the swine flu vaccination.
• Main reasons given for declining were safety concerns and being generally healthy.
• Believing the vaccine to be ineffective was strongly associated with declining it.

## Introduction

During the latter stages of the 2009/10 influenza A H1N1v (“swine flu”) pandemic, many governments offered the newly developed swine flu vaccine to members of their publics. Within the United Kingdom, the vaccine was offered to: people who fell into the usual seasonal flu vaccine at-risk groups as a result of having one of a range of chronic illnesses; pregnant women; household contacts of immunocompromised people; front-line health and social care staff; and children aged between 6 months and 5 years (Hine, 2010). Whilst frontline health and social care staff were offered the vaccine through their occupational health departments at work, other groups were offered it by the general practitioner [primary care practitioner], typically by letter. Despite an extensive media campaign aimed at maximising uptake, most people who were offered the vaccine declined it. According to official figures, the uptake rate amongst targeted patient groups was 34.5% (Sethi and Pebody, 2010). This low uptake was mirrored in countries world-wide, with a median coverage estimated at 21% (Brien and Kwong, 2011). Understanding why uptake was low is important if we are to improve vaccine coverage in the next pandemic.

Several factors may influence uptake. Recent systematic reviews of pandemic vaccine uptake (Bish et al., 2011, Brien and Kwong, 2011) have suggested that men, people from ethnic minorities and people with a chronic illness are most likely to be vaccinated, whilst evidence concerning the role of socioeconomic status and age is mixed. Within England, however, higher age appears to be associated with uptake (Biro, 2013). The psychological predictors of uptake that have been investigated broadly fit with the framework of Protection Motivation Theory (Rogers, 1975). This theory specifies that both ‘threat appraisals’ and ‘coping appraisals’ are important in determining health protective behaviour. In the context of a pandemic, threat appraisal relates to how one perceives the risk associated with pandemic flu. According to the theory, behaviours designed to protect health are more likely to occur if a risk is seen as being particularly likely to affect an individual and to have severe implications. Coping appraisal relates to how one perceives the behaviours that are available to protect against the threat, with a protective behaviour being more likely to be adopted if it is seen as effective (‘response efficacy’ in the terminology of Protection Motivation Theory), having few costs or side effects (‘response costs’) and being something that the individual is capable of carrying out (‘self efficacy’). During the swine flu pandemic, factors such as worry about the risk of catching the illness (a concept which fits broadly within the category of threat appraisal (Boer and Seydel, 1996)), perceptions about the severity of the pandemic and perceptions about the efficacy and safety of the vaccine have all been reported as predicting vaccine uptake (Bish et al., 2011, Brien and Kwong, 2011). Factors that do not fit neatly within the Protection Motivation Theory may also have some explanatory power, including previous acceptance of the seasonal flu vaccine (Bish et al., 2011, Brien and Kwong, 2011) and trust in official agencies (Siegrist and Zingg, 2014, Tucker Edmonds et al., 2011), a concept which, in turn, incorporates perceptions of the competence and honesty of those recommending vaccination.

A difficulty with many studies that have so far explored predictors of pandemic vaccination is their reliance on self-reported intention to be vaccinated as the outcome measure. Whilst intention to be vaccinated is a good predictor of subsequent vaccination, the association is not perfect (Lehmann et al., 2014, Sheeran, 2002). Studies assessing predictors of actual pandemic vaccination amongst the general public remain in the minority (Bish et al., 2011, Brien and Kwong, 2011). To our knowledge, none have been conducted in the UK which have examined pandemic-related perceptions as predictors (Bish et al., 2011, Brien and Kwong, 2011).

In this study, we conducted a secondary analysis of a dataset derived from a series of national telephone surveys conducted in the UK during the 2009/10 pandemic. These contained data on uptake of the swine flu vaccine amongst people who had been offered it by their primary care physician. We tested whether uptake was associated with variables relating to: demographic profile; worry and believing that too much fuss had been made about the pandemic (as components of threat appraisal); the perceived efficacy of the vaccine (as a component of coping appraisal); perceived preparedness of the government for a pandemic and satisfaction with the amount of information being provided about the pandemic (as components of trust); knowing people who had been affected by swine flu and having previously had the seasonal flu vaccine.

## Methods

### The surveys

A full description of the survey methods has been provided elsewhere (Rubin et al., 2015, Rubin et al., 2011, Rubin et al., 2015). In brief, during the course of the pandemic, 39 telephone surveys were commissioned by the English Department of Health and run by the Ipsos MORI Social Research Institute. Over time, survey questions were modified or removed and new questions were added to meet the department's changing priorities in understanding the reactions of the UK population to the ongoing pandemic. Sampling procedures and eligibility criteria were identical across all of the surveys. Random digit dialling and proportional quota sampling were used to ensure that the sample for each survey was demographically representative of the UK population. The quotas ensured that the number of participants within given groups for age, sex, geographical region and social grade (a classification system based on the occupation of the chief income earner of a household) were equivalent to the latest census statistics for the UK population. To be eligible for a survey, respondents had to be 16 years or over and speak English. Each survey was introduced to the participants as “a national survey on a variety of subjects.” Other topics were only asked about after all influenza-related questions had been covered. Response rates for each survey, calculated as the number of completed interviews divided by the total number of people spoken to, were in the region of 9% to 10%.

Distribution of the vaccine to general practices, and the time-lag involved in contacting people who were eligible to receive it and making appointments for them to be vaccinated, meant that the number of people vaccinated increased steadily over time. A visual inspection of the percentage of respondents who reported having received the H1N1 vaccination in each survey suggested that uptake had begun to plateau by late December 2009. We assume that by this stage, practical issues relating to the initiation of the vaccination campaign were no longer the main barriers to uptake. To provide a larger sample size for our analyses, we therefore pooled the data from the final five surveys, spanning 28 December 2009 to 14 February 2010, for our analyses. In total, 5290 people completed these surveys. All individual-level data for all of these participants were available on a single spreadsheet, allowing this analysis to take place.

### Vaccine uptake

All the participants were asked “have you been invited by your GP [general practitioner]'s surgery to have the swine flu vaccination, or not?” Possible answers were “yes”, “no” or “don't know”. The participants were later informed that “the swine flu vaccination programme has started” and were asked to “please tell me whether you have had the swine flu vaccine, or if not, how likely, if at all, are you to take up a swine flu vaccination if offered it?” Possible answers were “I have already had the vaccination”, “very likely”, “fairly likely”, “not very likely”, “not at all likely” and “don't know”. The participants who reported being “not very” or “not at all likely” to have the vaccination were asked an open-ended follow-up question: “what would you say is the main reason why you would not be likely to take up a swine flu vaccination if offered it?”

### Demographic characteristics

Demographic data recorded for each participant included their age, sex, ethnicity, parental status and social grade (using the categorisation of ‘ABC1’ [broadly managerial or professional] vs ‘C2DE’ [broadly manual or casual workers or unemployed on state benefit]). (MRS, 2006). The participants were asked “how is your health in general” (‘very good,’ ‘good,’ ‘fair,’ ‘poor,’ ‘very poor’) and “do you have any long-standing illness, disability or infirmity?”

### Variables relating to threat appraisal, coping appraisal, seasonal flu vaccination and trust

In terms of variables that might map onto threat appraisal, the participants in the surveys were asked “how worried, if at all, would you say you are now about the possibility of personally catching swine flu?” The permitted responses were “very worried,” “fairly worried,” “not very worried,” “not at all worried” and “don't know”. The participants were also asked whether they agreed or disagreed that “too much fuss is being made about the risk of swine flu.”

With respect to coping appraisal, participants in the final three surveys were asked to rate their opinions on the effectiveness of the swine flu vaccination in “reducing the risk of catching or passing on swine flu”. Answers were recorded on a scale of 1–10, with 1 being “no difference” and 10 being “it is vital”.

The participants in the final four surveys were “have you had the regular winter flu jab in previous years, or not?”

The participants were asked whether anyone in the following groups had had swine flu: themselves; their children; or friends, colleagues or other family members. In the first three surveys, they were given ‘yes’ or ‘no’ options for each category. For the final two surveys, a ‘not sure’ option was also offered.

Two questions related to official responses to the pandemic that might reflect trust. First, the participants were asked “how well prepared do you think the Government is for a swine flu pandemic,” with possible responses being ‘very,’ ‘fairly,’ ‘not very’ and ‘not at all.’ Second, the participants were asked “how satisfied or dissatisfied are you with the amount of information available to you on swine flu, from any source,” with possible responses being ‘very satisfied,’ ‘fairly satisfied,’ ‘neither satisfied nor dissatisfied,’ ‘fairly dissatisfied’ and ‘very dissatisfied.’

### Analyses

We excluded the participants who had not been invited to have the swine flu vaccine by their GP. We also excluded the participants who had not yet accepted an invitation but who reported that they were ‘very’ or ‘fairly’ likely to be vaccinated in the future, on the basis that these responses reflected intentions rather than behaviour.

The responses to the open-ended question regarding reasons for not being vaccinated were initially coded by interviewers working for Ipsos MORI, using 38 categories that were pre-selected as potentially important by the Department of Health in liaison with Ipsos MORI. To facilitate reporting, we grouped some of these categories together.

For the quantitative questions, we counted responses of ‘don't know’, ‘unsure’, ‘not applicable’ or ‘neither agree nor disagree’ as missing data. Because we had low sample sizes for several analyses, we combined some response options for some predictor variables (see Table 1, Table 2 for details). Binary logistic regressions were used to identify significant predictors of vaccine uptake. A first set of regressions assessed the role of demographic variables in predicting the likelihood of vaccine uptake, including after adjustment for all other demographic variables. A second set of regression models then tested the association between the remaining predictor variables and vaccine uptake whilst adjusting for demographic characteristics. Because not every question was used in every survey, some of our analyses were based on a subset of the surveys.

## Results

A total of 1320 participants had been invited by their GP to have the swine flu vaccination. Of these, 734 (55.6%) had received it. The remaining 586 participants included 127 (9.6%) who reported being very likely to be vaccinated, 50 (3.8%) who were fairly likely to be, 96 (7.3%) who were not very likely, 300 (22.7%) who were not at all likely and 13 (1.0%) who did not know. Our analyses therefore focussed on the 734 participants who had been vaccinated and the 396 who had not been and were unlikely to be.

### Qualitative data about uptake of the swine flu vaccine

The top answers given by people as their main reason for not being vaccinated were: I am concerned over how safe the vaccine is/it hasn't been tested enough (101 people out of 396 asked, 25.5%); I am generally healthy and not overly concerned about catching swine flu (*n* = 96, 24.2%); I don't like having vaccinations (*n* = 36, 9.1%); I don't know enough about the vaccine (*n* = 22, 5.6%); and side effects/it makes you ill/it causes flu or a bad reaction (*n* = 17, 4.3%).

### Association between demographic characteristics and likelihood of being vaccinated

The demographic characteristics of the participants and the associations between these characteristics and vaccine uptake are shown in Table 1. The participants were less likely to be vaccinated if: they were female, had children aged 16 or under, were aged 25 to 34, had good or very good health, and had no long-standing infirmity or illness. After adjusting for all other demographic variables, only the relationships with sex and with the presence of a long-standing infirmity or illness remained significant. The inclusion of all demographic variables in the model resulted in a classification accuracy of 64.9% and a Nagelkerke R^2^ of 0.05.

### Association between threat appraisal, coping appraisal, seasonal flu vaccination, knowing someone who had contracted flu and trust, with vaccine uptake

Table 2 shows the results for the non-demographic predictor variables and their associations with vaccine uptake. The participants were less likely to be vaccinated if they: felt that too much fuss was being made about the risk of swine flu, felt that the vaccine was ineffective in preventing the spread of swine flu, or had not had the seasonal flu vaccine in previous years. These associations remained significant after adjusting for demographic characteristics. The inclusion of all variables in Table 2 in addition to all demographic variables in the model improved the classification accuracy to 84.5% and a Nagelkerke R^2^ of 0.78.

## Discussion

According to our data, acceptance of the swine flu vaccine amongst adults offered it by their primary care practitioner was 55.6%. This figure is higher than the official figure of 34.5% which was generated by gathering data directly from primary care practices (Sethi and Pebody, 2010). Overestimation of vaccine uptake appears to be a general problem with telephone surveys (Brien and Kwong, 2011). Several explanations may account for this, most particularly selection bias (with people who are willing to take part in a survey being more compliant with healthcare recommendations than those who decline to take part) and recall bias (with participants who have been vaccinated being more likely to remember receiving an invitation from their GP than those who have not been vaccinated). Although substantial advantages exist to using rapid turnaround telephone or web-based surveys to track behaviour and concerns during a major public health incident (Rubin et al., 2008), caution is required in interpreting data derived from such methods.

Despite this caveat, our data support the role of several demographic variables in predicting vaccine uptake. Women, people with better self-rated health, those without a long-lasting illness or infirmity, those in the 25 to 34 age bracket and those with young children were all less likely to be vaccinated. Many of these findings have been reported previously (Bish et al., 2011, Brien and Kwong, 2011). Once all other demographic variables were adjusted for, however, only sex and the presence of long-lasting illness or infirmity remained significant. Having a chronic illness may increase perceptions of vulnerability to flu. Several chronic illnesses were also risk factors that determined whether someone would be offered the swine flu vaccine. It is therefore logical that the presence of a chronic illness could explain the association between self-reported health, age or having young children (itself associated with age) and vaccine uptake. The reasons why men are more likely to accept the vaccine than women remain opaque, with previous research having found no major differences between men and women in terms of their motives for not accepting the swine flu vaccination (Velan, 2011). Others have hypothesised that, for seasonal flu vaccine, low uptake amongst women may result from healthcare provider bias, a tendency for women to be more likely to see healthcare providers who are less encouraging about the vaccine than men, or a tendency for women to be less receptive to recommendations from healthcare providers (Jiménez-García et al., 2010). Whether these factors are relevant during a pandemic is unknown.

The importance of a person's health in determining vaccine uptake also accords with the qualitative data for the open-ended question included in the surveys. Here it was striking that only two factors accounted for the majority of reasons given for not being vaccinated: being fit and healthy, and believing that the vaccine was unsafe. Yet whilst other reasons were rarely spontaneously mentioned by the participants, direct questioning did reveal the importance of additional factors. By far and away the strongest of these was a prior history of vaccination against seasonal flu. This is in line with multiple previous studies (Bish et al., 2011, Brien and Kwong, 2011) and is likely to reflect several underlying issues, including the role of habit, previous positive experiences with vaccination, the absence of barriers to being vaccinated and similarities between the seasonal and swine flu vaccine in terms of perceptions about their efficacy, concern about the effects of flu and trust in GPs.

As predicted by the Protection Motivation Theory (Rogers, 1975) and in line with several previous studies (Bish et al., 2011, Brien and Kwong, 2011), the perceptions about the efficacy of the swine vaccine strongly predicted uptake. The role of variables related to threat appraisal was less clear. Earlier studies have suggested that anxiety or worry about contracting swine flu were associated with a higher likelihood of being vaccinated (Bish et al., 2011, Brien and Kwong, 2011, Rubin et al., 2011). In the present analyses, however, worry about the possibility of catching swine flu was not associated with vaccination. This apparent discrepancy may be an artefact caused by the timing of the questions in the surveys. If worry motivates people to be vaccinated, then a corollary is that being vaccinated should reduce worry removing any statistical association. In contrast to worry, perceiving that too much fuss had been made about the pandemic was associated with not being vaccinated. Perceptions of “too much fuss” may be a useful measure of threat appraisals in this context, as they reflect a participant's assessment of how severe the pandemic is in general, something that should not be affected by the act of receiving a vaccination.

Although hypothesised by others as being relevant in predicting vaccination (Tucker Edmonds et al., 2011) and having been shown to predict other protective behaviours during the 2009/10 pandemic (Rubin et al., 2009a), our trust-related variables showed no association with vaccination status. These variables were not ideal as indicators of trust, however, and caution is required in their interpretation. First, the questions available to us tapped only two variables that make up the broad construct of trust: the perceived competence of the responding agency and their perceived openness. Other variables may also be important, including the perceived priorities of the responding agency and the consistency of their messages (Peters et al., 1997, Rubin et al., 2012). Second, the questions were not specific to the vaccine, but rather to the general handling of the pandemic. Third, the question addressing satisfaction with the amount of information available related to information from any source rather than from the government. And fourth, it is possible that trust in the government in general is less important in predicting vaccination than trust in specific responding agencies such as the NHS, one's primary health practitioner or even the vaccine manufacturers. Additional research on a wider array of trust-related variables is warranted.

### Limitations

In addition to the issues raised above, several additional caveats should be borne in mind when evaluating our results. First, the questions used in the original surveys were not always ideal for our purposes, reflecting the pragmatic considerations at the time and the speed with which the surveys had to be put into the field. Opportunities were missed to assess additional constructs that have been specified by theories of behaviour change (for example, self-efficacy) and to test the wording of questions (for example, the complex ‘worry’ item). Recent work by our team in tandem with UK stakeholders has resulted in a new set of survey items which will hopefully resolve some of these issues in any future pandemic (Rubin et al., 2014).

Second, low sample sizes for some of our analyses resulted in wide confidence intervals and it is possible that as a result some important associations were not identified as significant.

Third, a general problem with all research based on the 2009/10 pandemic is its generalisability to future pandemics. The relatively mild nature of swine flu may have led many members of the public to equate it to seasonal flu. This may not be the case in a more severe pandemic or one involving a different epidemiological pattern (for example, where children are disproportionately affected). In such situations, both the level of uptake of a vaccine and the determinants of uptake may prove to be different.

Finally, because we analysed multiple predictor variables, there is a possibility that some of our significant findings are spurious, Type I errors.

### Conclusions

Uptake of the 2009/10 swine flu vaccine in the UK was poor amongst those offered it by their primary care physician. Low uptake was affected by perceptions that it was unnecessary for people who were ‘fit and healthy’ to be vaccinated, that the vaccine was unsafe or insufficiently tested, that the vaccine was ineffective and that too much fuss was being made about the pandemic. Tackling these perceptions may be the key to encouraging uptake in future pandemics.

## Contributorship statement

All authors contributed to the conception, analytic plan and write-up for this manuscript. Julia You-Kyung Han wrote the first draft, which was subsequently revised by all authors. All authors approved the final version.

## Conflict of interest statement

The authors declare that there are no conflicts of interest.

## Figures and Tables

**Table 1:** Demographic characteristics of the participants and the association between these characteristics and vaccine uptake in the UK during 2009/10. Adjusted odds ratios were adjusted for all other demographic variables: age, sex, ethnicity, parental status, social grade, general health status and presence of any long-standing infirmity or illness.

Variable	Levels	Vaccinated (*n* = 734)	Not vaccinated (*n* = 396)	Odds ratio (95% confidence interval) for association with vaccination status	Adjusted odds ratio (95% confidence interval) for association with vaccination status
Gender	Male	322 (69.1%)	144 (30.9%)	1.37 (1.06 to 1.76)	1.34 (1.03 to 1.73)
	Female	412 (62.0%)	252 (38.0%)	Reference category	Reference category
Age	16 to 24	19 (70.4%)	8 (29.6%)	1.20 (0.52 to 2.80)	1.44 (0.60 to 3.46)
	25 to 34	26 (51.0%)	25 (49.0%)	0.53 (0.30 to 0.94)	0.73 (0.38 to 1.38)
	35 to 54	167 (62.1%)	102 (37.9%)	0.83 (0.61 to 1.12)	0.96 (0.66 to 1.39)
	55 to 64	164 (67.2%)	80 (32.8%)	1.04 (0.75 to 1.43)	1.02 (0.73 to 1.42)
	65 or older	358 (66.4%)	181 (33.6%)	Reference category	Reference category
Social grade	ABC1	333 (61.1%)	203 (37.9%)	0.79 (0.62 to 1.01)	0.86 (0.66 to 1.11)
	C2DE	401 (67.5%)	193 (32.4%)	Reference category	Reference category
Ethnicity	White	702 (64.6%)	384 (35.4%)	0.69 (0.35 to 1.35)	0.69 (0.34 to 1.39)
	Other ethnicity	32 (72.7%)	12 (27.3%)	Reference category	Reference category
Parental status	Has child 16 yrs. or under	97 (57.4%)	72 (42.6%)	0.69 (0.49 to 0.96)	0.83 (0.54 to 1.27)
	Has older child or no children	637 (66.3%)	324 (33.7%)	Reference category	Reference category
General health status	Poor or very poor	123 (73.7%)	44 (26.3%)	1.69 (1.26 to 2.28)	1.33 (0.96 to 1.84)
	Fair	210 (71.4%)	84 (28.6%)	1.89 (1.30 to 2.76)	1.33 (0.87 to 2.02)
	Very good or good	396 (59.6%)	268 (40.4%)	Reference category	Reference category
Presence of any long-standing infirmity or illness	Yes	450 (71.4%)	180 (28.6%)	1.90 (1.48 to 2.44)	1.60 (1.20 to 2.12)^a^
	No	280 (56.8%)	213 (43.2%)	Reference category	Reference category

a. Re-analysis of this association excluding “general health status” as a covariate had little impact on the association (aOR 1.82, 95% CI (1.41, 2.34).

**Table 2:** Perceptions about swine flu, the vaccine and official responses to the outbreak, and their association with vaccine uptake in the UK during 2009/10. Adjusted odds ratios were adjusted for age, sex, ethnicity, parental status, social grade, general health status and presence of any long-standing infirmity or illness.

Variable	Levels	Vaccinated (*n* (%) unless specified)	Not vaccinated (*n* (%) unless specified)	Adjusted odds ratio (95% confidence interval) for association with vaccination status
Worry about the possibility of personally catching swine flu	Very or fairly worried	64 (70.3%)	27 (29.7%)	1.18 (0.73 to 1.92)
	Not very or not at all worried	666 (64.5%)	367 (35.5%)	Reference category
Too much fuss is being made about the risk of swine flu	Disagree	275 (74.9%)	92 (25.1%)	2.12 (1.51 to 2.70)
	Agree	376 (58.7%)	265 (41.3%)	Reference category
I have had swine flu.	Yes	19 (59.4%)	13 (40.6%)	0.84 (0.40 to 1.77)
	No	715 (65.1%)	383 (34.9%)	Reference category
My children have had swine flu (analyses restricted to parents).	Yes	9 (69.2%)	4 (30.8%)	1.54 (0.42 to 5.74)
	No	88 (56.4%)	68 (43.6%)	Reference category
Friends, colleagues or other family members have had swine flu	Yes	167 (62.5%)	100 (37.5%)	0.95 (0.70 to 1.29)
	No	567 (65.7%)	296 (34.3%)	Reference category
Perceived efficacy of the vaccine	Ratings of 1 (makes no difference at all) to 10 (it is vital)	Median: 10 (range 1 to 10, *n* = 441)	Median: 5 (range 1 to 10, *n* = 229)	1.67 (1.55 to 1.81)
Have you had the seasonal flu vaccine in previous years?	Yes	505 (76.9%)	152 (23.1%)	6.77 (4.74 to 9.67)
	No	83 (34.9%)	155 (65.1%)	Reference category
How well prepared is the Government for a swine flu pandemic?	Very or fairly prepared	553 (66.1%)	283 (33.9%)	1.34 (0.98 to 1.84)
	Not very or not at all prepared	146 (62.4%)	88 (37.6%)	Reference category
Satisfaction with amount of information available	Very or fairly satisfied	639 (66.5%)	322 (33.5%)	1.63 (0.97 to 2.75)
	Very or fairly dissatisfied	42 (60.0%)	28 (40.0%)	Reference category
